# Reflections on a project to prevent suicide and self-harm among prisoners identified as high risk in two prisons in Northern England

**DOI:** 10.1186/s40352-018-0080-7

**Published:** 2018-12-03

**Authors:** Paul Biddle, Wendy Dyer, Richard Hand, Charlitta Strinati

**Affiliations:** 1 Department of Social Sciences, Northumbria University, Newcastle upon Tyne, NE1 8ST UK; 2 Tees Esk & Wear Valley NHS Foundation Trust, Roseberry Park Hospital, Middlesbrough, TS4 3AF UK

**Keywords:** Suicide, Self-harm, Prison, Prisoners, Staff-led innovation

## Abstract

**Background:**

This article critically explores the implementation and evaluation of a project designed, delivered and evaluated by frontline staff to improve prison responses to prisoner suicide and self-harm. We begin by evidencing the need for the project and detail its content, delivery and attempts at evaluation. We draw on the reflections of the three practitioners most closely involved in its development, delivery and review in order to explore lessons learned for future staff-led projects including those aimed at tackling prison suicide and self-harm.

**Results:**

Findings from staff reflections suggest that the development, implementation and evaluation of the project were influenced by a combination of issues around: project focus, communication and professional relationships, the institutional environment, funding and time, roll-out and evaluation, and the need for a ‘champion’ role.

**Conclusions:**

There is limited evidence that the project left a modest positive legacy in terms of impact. A more substantial legacy of the project is provided in terms of actionable learning points for future projects of this nature.

## Introduction

This article discusses and reflects on a staff-led project designed to reduce levels of suicide and self-harm in one adult male Category ‘A’ prison and one Young Offenders Institution (YOI) in England. The project attempted to reduce the risk of suicide and self-harm by developing the confidence of prison staff working with prisoners in distress or struggling with mental health difficulties, and by providing in-cell therapeutic interventions for prisoners. The project was delivered between November 2015 and September 2016 by NHS Foundation Trust frontline staff, via grant funding from the Ministry of Justice (MoJ).

## Background

High levels of suicide and self-harm have been longstanding problems for the English prison system (Barker et al., 2014; Hawton et al., 2014; Humber et al., 2011; Patry & Magaletta, 2014; National Audit Office, 2017). There were 70 apparent suicides in 2017 (17% fewer than recorded the previous year, but still historically high). In 2017, incidents of self-harm reached a record high, with a record 42, 837 incidents recorded, representing an increase of 12% from 2016. Furthermore, incidents of suicide and self-harm, per 1000 male prisoners, remain higher than a decade ago (Kaster et al., 2017; Ministry of Justice, 2017a; Ministry of Justice, 2018). Explanations for suicide and self-harm appear complex and to be a result of the interaction of historic, personal, medical and situational factors. (Daniel, 2006; Dear, 1999; Kaster et al., 2017; Maranzo et al., 2016; Miller & Najavits, 2012; Timmerman & Emmelkamp, 2001; Wolff et al., 2014).

Environments and interventions that appear able to reduce prisoner suicide and self-harm risk include those creating opportunities for positive interactions and relationships between staff and prisoners. Maximising the time prisoners spend out of cell undertaking purposeful activities is also important to reduce rates of suicide and self-harm (Biggam & Power, 1998; Barker et al., 2014; Hawton et al., 2014; Leese et al., 2006; Maranzo et al., 2016; Maranzo et al., 2012). ‘Listener’ and ‘Buddy’ schemes, provided by trained prisoners may also help those at risk of suicide and self-harm (Foster, 2011). The value of therapeutic educational and vocational activities to reduce prisoners risk of self-harm and suicide, linked to physical, sensory and social isolation, has been recognised (Barker et al., 2014; Daniel 2006; Konrad et al., 2007). The potential value of creative activities, alongside relaxation and mindfulness meditation has also been discussed (World Health Organisation, 2007; Rethink Mental Illness, 2014). Relaxation and mindfulness activities can improve sleep and lower anxiety, with yoga improving mood and reducing anxiety, stress, anger, aggression and impulsive behaviour (Bilderbeck, et al., 2013, Shapiro & Cline, 2008; Lutz, 1990; Yoshihara et al., 2011). Better training, support and supervision for prison staff can improve attitudes, resulting in responses that are more effective at managing suicide and self-harm risk (Maranzo et al., 2012). Evidence indicates the potential value of adopting Trauma Informed Care-models that attempt to identify and respond to historical and unresolved trauma’s that contribute to a prisoner’s current mental health problems (Havenger, 2010; Miller & Najavits, 2012; National Resource Centre for Justice Involved Women, 2013). There is also a need to ensure that frontline prison officers have the skills to respond to incidents of self-harm and suicide as it they who are often required to initially respond to incidents as they are in closest proximity (Ramluggun, 2011).

To respond to the multiplicity of interrelated factors that increase risk of suicide and self-harm, policy and practice has sought to develop an integrated approach to reduce the incidence of both in the prison population. The approach attempts to improve screening, identification and observation, improve interventions and inter-disciplinary working, modify the physical prison environment and promote staff training (Humber et al., 2011; Konrad et al., 2007). Prison Service Instruction 64/11 (PSI 64/11) aims to manage those at risk of harming themselves, the roll out of a reception-screening tool aims to identify suicide and self-harm risk, and the use of the Assessment, Care in Custody and Teamwork (ACCT) procedure aims to improve the quality of care by introducing individualised, flexible, multi-professional care-planning, supported by improved staff training in case management and by improving ongoing risk assessment of prisoners (Ministry of Justice, 2013). PSI 64/11 states relevant parties should be invited to attend ACCT reviews in order to collaboratively make decisions around the prisoner’s level of risk and the management of this. Attendees can include prison wing staff, prison safer custody staff, mental or general health care workers, chaplaincy and Independent Monitoring Board representatives. PS1 64/11 makes reference to building positive engagement and relationships between prisoners and staff which should be facilitated within the prisons by each prisoner having a personal officer. PSI 64/11 also outlines some mental health terminology, risk factors and potential for self-harm and suicide, and aims to improve understanding of different conditions to help staff working with prisoners who experience such difficulties.

Mental Health In-Reach teams have also been introduced to identify and treat mental disorders among prisoners (Steel et al., 2007). Peer support interventions and listening schemes have also been developed as have Safer Cell/Custody schemes and first night centres to manage suicide and self-harm risk (Barker et al., 2014; Daigle et al., 2007; Humber et al., 2011). There has been investment in drug treatment provision, which is crucial as suicide and self-harm risk can be elevated by substance withdrawal (Crighton & Towl, 1998; Shaw et al., 2004).

Despite this body of research, policy and practice, concerns remain that existing approaches are not sufficiently effective at reducing suicide and self-harm (PPO, 2016; PPO Annual Report, 2014; PPO Annual Report, 2015). It has been argued that the ACCT process cannot operate effectively in an environment characterised by large prisoner population and reduced staff levels (PPO, 2017). The ACCT process does not always incorporate standardised processes for assessing the risk of repeat self-harm, with some prison staff unsure of how to assess risk appropriately. There are concerns that follow up and support for prisoners following closure of their ACCT is limited (Humber et al., 2011; PPO Annual Report, 2015; Steel et al. 2007) and Brooker et al. (2010) argue that Mental Health In-Reach teams have been stretched and challenged by a blurring between primary and secondary care and under resourcing. There is also recognition that the constant supervision policy may be potentially problematic as it may have negative impacts on prisoners at risk of harming themselves (Ministry of Justice, 2012; Sakinofsky, 2014). The aforementioned levels of suicide and self-harm appear to evidence the limitations of the current policy and practice.

Research exploring how best to tackle prisoner suicide and self-harm has been compromised by insufficiently reliable and robust monitoring systems, an inability to generalise findings because of unique features of prisons participating in specific studies, and a reliance on prisoner perceptions/feedback (Barker et al., 2014; Humber et al., 2011; Naud & Daigle 2013). Additional problems include difficulties securing effective implementation in the prison environment, small sample sizes, attrition rates and missing data (Barker et al., 2014; Eccleston & Sorbello, 2002; Hayes et al., 2008; Humber et al., 2011; Naud & Daigle, 2013).

### The project

#### Drawing on experience

*“PSI 64/11 works well in principle, however experience suggested prisoners are often reluctant to go to officers for support and find alternative ways to manage”* (*interviewee 2*).

*“Experience of working in prison settings suggested that the majority of staff know little about mental health and personality disorders that prisoners they work with may be experiencing because front-line staff will not have access to the relevant information”* (*interviewee 2*).

and on interventions that appear to reduce suicide/self-harm risk discussed, this project sought to reduce levels of self-harm and suicide in prisons by: 1) developing staff ability and knowledge about suicide, self-harm and mental health; and 2) the provision of practical, therapeutic in-cell activities for prisoners. This project was a service development opportunity as outlined by the requirements of the MoJ funding competition. Support for the implementation of the project was obtained from the MoJ, Her Majesty’s Prison and Probation Service (HMPPS), individual Prison Governors, and the NHS Trust Offender Health Governance board. Ethics permission was obtained by University staff from the University Ethics Committee to conduct interviews with practitioners involved with the project in order to inform this publication.

The project was delivered at two English prisons, selected because of their more stable populations:

- A High Security prison holding 800 category A and B sentenced and category A remand male prisoners with the majority serving lengthy or indeterminate sentences for very serious offences. Levels of self-harm at this institution are generally low and occur generally among a small number of prisoners. On average, 14 prisoners a month were subject to ACCT case management because they were at increased risk of self-inflicted death or self-harm (Her Majesty’s Inspectorate of Prisons).
- A Young Offender Institution (YOI) that that can hold 513 male prisoners aged between 18 and 21. There are an average of 17 incidents of self-harm a month involving an average of seven prisoners at this YOI. On average, 16 ACCT case management documents a month are opened for prisoners at risk of self-inflicted death and self-harm (Her Majesty’s Inspectorate of Prisons).^1^

The project was funded by a grant from the MoJ and delivered by an NHS Foundation Trust. It operated from November 2015–September 2016. The project was developed and co-ordinated by an offender health service manager, a Psychologist and an Assistant Psychologist. The project was led initially by a Modern Matron. The Safer Custody Leads of each establishment were identified as points of contact within the prisons to co-ordinate delivery as they could potentially act as a link between the project team and wider prison. They are prison staff responsible for “delivering Safer Custody procedures and practices to ensure prisons are safe places (Ministry of Justice, 2016). The project comprised:

### Staff development activities

In order to understand prison staff training needs, a mental health-focused training needs assessment questionnaire was developed by the project team. This was disseminated by prison staff and completed by 22 members of prison staff across both institutions with safer custody responsibilities. This resulted in the project Assistant Psychologist, developing e-learning packages for Attention Deficit Hyperactive Disorder, self-harm, Obsessive Compulsive Disorder, Personality Disorder, which were shared with Safer Custody Leads, which included knowledge tests. These e-learning packages were created specifically in response to feedback from prison staff and also to developments within the NHS Foundation Trust around trauma informed practice. The packages were based on those developed by the Trauma-Informed Care Lead within the NHS Foundation Trust and tailored to meet the needs of prison staff. Other training options were explored such as the Knowledge and Understanding Framework for Personality Disorder (KUF). However, the aim of this part of the project was to provide information to as many staff as possible, as quickly as possible and other existing training is provided over a number of days or longer.

A range of electronic and hard-copy ‘Prison Staff Guides’ were also created and given to each wing in each institution to help staff respond to prisoners experiencing and displaying difficulties associated with risk of suicide or self-harm. These guides covered a wide range of issues including anger, bereavement, anxiety, panic attacks, loneliness, low self-esteem and sleeping problems. The guides were promoted in an edition of the Safer Custody Newsletter. Personal visits were made by the project Assistant Psychologist to both institutions to inform Safer Custody staff about the guides. Prisoners staffing the Prison Information Desks also received paper copy resources. In addition, an electronic document was created that included useful online resources and hyperlinks that all Prison Staff can click to access information on a wide range of mental health conditions. All of this activity was designed to empower the Prison Staff to intervene appropriately responding to prisoners in distress who may not meet the criteria for an immediate intervention by the Mental Health Team, or who may need support outside the hours of Mental Health Team operation.

### Trauma informed service

The initial project included plans to introduce a full Trauma Informed Service at the High Security prison – on the basis that this was the more ‘settled’ establishment, where such an approach could therefore be more speedily and consistently implemented than was possible at the YOI. However, the duration of the project (and funding) meant this proved impracticable. Inclusion of a full trauma informed service was an initial requirement of the MOJ funders and therefore the project proposal included plans to introduce a full Trauma Informed Service at the High Security prison. However from the outset both the funder and NHS foundation Trust recognised this was unfeasible due to the limited budget and timescale. It was therefore decided – by both the project team and the MoJ – to focus on awareness-raising activities and training for prison staff, designed to develop a more trauma-informed prison culture. Two face-to-face awareness training sessions were delivered with members of prison staff selected by the Safer Custody Lead (including Safer Custody Officers). The first session was delivered by the institution’s Trauma Informed Care Lead, the second was jointly delivered by the project Assistant Psychologist and an additional Assistant Psychologist. A total of 15 staff attended. The training package included information about trauma, the impact it may have on prisoners and their behaviour, plus advice about how to work in a trauma informed way. It was envisaged that staff attending the training would both informally cascade the knowledge of a Trauma Informed Service to colleagues because of changes, resulting from the training, to their way of thinking and behaving in relation to prisoners; and also formally signpost other staff to the training and resources that were available. To complement this training, a trauma informed e-learning package for prison staff was developed. This was a summarised version of the awareness training delivered and included knowledge quizzes to test understanding, and was uploaded to the IT network so all staff could access it. The e-learning package was promoted via the Safer Custody Newsletter, emails to key staff within Safer Custody and via the attendance of a member of the project team at morning briefings which take place on individual prison wings.

### Therapeutic in-cell activities for prisoners

Therapeutic In-Cell Activities were developed, based on elements of Dialectical behaviour therapy (DBT), including mindfulness and distress tolerance (distraction). All resources, including paper-based resources, were developed with the recipients in mind. Therefore prisoner resources were self-help resources and prison staff received resources aimed at providing skills in working with prisoners in distress. The therapeutic activities for prisoners comprised:

- **Big Orange Boxes** (or Bob Boxes) available to prisoners subjected to the ACCT process. Three Bob Boxes were provided to the High Security prison and two were provided to the YOI. Bob Boxes were developed to help prisoners during periods of anxiety, distress and agitation. Bob Boxes were introduced to both prisons staff via focus groups (attended by Safer Custody Governors, Custody Managers and Senior Officers with Safer Custody-related roles). Roll-out was coordinated by the Safer Custody Leads at each prison who were responsible for making boxes available to prisoners as needed, based on a process map developed by the project team to facilitate appropriate roll-out. The Bob Boxes contained drawing and art activities, water colour pencil pack, playing cards, Rubik’s cube, ‘tumbling tower’ blocks, stress balls, a yoga/relaxation pack, a paper craft/origami pack and a CD player, with CDs. The Bob Boxes also included leaflets for prisoners detailing the potential benefits of each of the resources, with guidance about how to use the resources where necessary, plus ‘Prisoner Profiles’ which prisoners could use to identify and record their own individual triggers and calming techniques and share with staff to inform ongoing care-planning. Prisoners using the Bob Boxes were also to be given an evaluation form by prison staff to evaluate the usefulness of the resources they had used.
- **Yellow Box Files** were developed (three per institution) for prisoners not subject to the ACCT procedure. These yellow box files included a playing card pack, two sets of colouring pencils, yoga leaflets, creativity leaflets, relaxation leaflets, creative writing pack, drawing packs and colouring packs. These were to be kept on the wings and made available to all prisoners upon request. The distribution of these files was co-ordinated by Safer Custody Teams.
- **Prescribed Relaxation Packs** were developed at the YOI only because the mental health team here had greater capacity to manage this intervention compared with teams at other prisons. The packs were developed for any prisoner with sleep disturbance (either self-reported or observed by staff), and contained a leaflet providing information on relaxation exercises, a guided relaxation CD, a yoga book, a stress ball and (optional) aromatherapy. The pack also included a diary sheet so prisoners could record their relaxation practice. Many of the packs resources were provided by the Phoenix Prison Trust which provides yoga and relaxation resources for all prisoners free of charge. Implementation was overseen by the Mental Health Team at each prison. Prisoners meeting the inclusion criteria had an initial screening appointment with a member of their Mental Health Team. The screening was informal and designed to explore motivation and identify any contraindications. Subject to a positive screening, each prisoner received a leaflet about relaxation and its benefits, which the Mental Health Team member explained to ensure the prisoner understood how to use all the resources in the pack. Prisoners were encouraged to carry out relaxation every day over the four-week period and record it in their relaxation diary. A member of the team met with the prisoner once a week to monitor progress and gage motivation.

These activities delivered were chosen as they reflected MoJ funding criteria, the knowledge and expertise of the project team and a review of good practice, but also because the project team was keen to further develop relationships between mental health and safer custody teams, and activities that could improve the delivery of ‘constant watches’:

*“There was some interest from the Ministry of Justice and a tender opportunity for things to use in prisons that would alleviate self-harm” (interviewee 3).*

*“I know that we had to have a look at how we interfaced, as mental health, with safer custody…improving communication…constant watch….looking at that process. Could we offer distraction, could we offer some self-help?” (interviewee 2).*

The project evolved over time, changing as a result as a result of discussions within the project team, wider prison staff, a review of existing evidence and the personal knowledge and experience of project staff:

*“It was evolving as we discussed more with the prisons. We did focus groups, we looked at what research is out there, what does work. We looked a Knowledge Transfer Partnership (KTP) that had happened at (name of prison) where they’d reduced self-harm. I was involved in the KTP so I knew that…and national guidance” (interviewee 2).*

### The project evaluation - methodology and findings part 1

The project evaluation was developed and implemented by the Mental Health Trust Project Team. It used a mixed-method approach. A feedback questionnaire was used to capture staff views of the Trauma Informed Service training. Eight participants completed this survey. The paper and online materials developed for staff were not evaluated.

The impact of the Bob Boxes was captured via administration of feedback forms to prisoners, interviews with prisoners, and feedback forms administered to staff. No data was provided by the High Security prison about the number of prisoners who used the Bob Boxes, with 16 prisoners using them at the YOI. Feedback forms were completed by a total of 10 prisoners and 7 prisoners participated in an interview. Eight staff completed BOB box feedback forms (three at the High Security prison and five at the YOI). No feedback was gathered about the impact of the yellow box files.

The prescribed relaxation component of the pilot was evaluated using the Patient Health Questionnaire (PHQ-9) (to measure levels of depression), the Generalised Anxiety Disorder (GAD-7) (used as a measure of anxiety) and the Warwick-Edinburgh Mental Well-being scale (WEMWBS) (to measure overall wellbeing). The measures were administered by the Trust Mental Health Team pre-intervention and at 4 weeks post-intervention. Prisoners also received a feedback questionnaire, contained in the pack, to complete about their experience. A total of 9 prisoners at the YOI completed the pre-intervention GAD-7, PHQ-9 and the WEMWBS, with 6 completing these 4 weeks post-intervention. No prisoners completed the feedback form as they were transferred to another establishment before they could do so, thus preventing follow-up.

The project findings relating to the trauma informed practice training suggests that it improved the confidence of trainees understanding of what a trauma informed service looks like. Feedback from prisoners who used the in-cell Bob Boxes was generally positive, with prisoners reporting that Bob Boxes helped them to manage distress, distract and calm them. Those prison staff who took part in the evaluation reported being confident in their knowledge of the Bob Boxes. GAD-7 and PHQ9 scores suggest that the prescribed relaxation resources had positive results – with scores lower on average after this intervention than before. The PHQ-9 score were lower post intervention for half (n-3) of respondents completing the tool, higher for 2 participants and unchanged for one. Prescribed relaxation generally appeared to be helpful in decreasing prisoner depression and anxiety, although this was not the case for all prisoners. However, only nine prisoners participated in prescribed relaxation, and only six participated in the four-week follow-up. Only a small number of prisoners completed feedback surveys (n-10) or interviews (n-7) about their use of Bob Boxes. Eight members of prison staff provided survey feedback. As no data was captured about overall Bob Box usage, it is impossible to determine the representativeness of findings. As discussed, electronic and paper resources for staff were not evaluated, and nor were the yellow box files of materials for prisoners.

While findings suggest the potential of the project to have a positive impact, the Mental Health Trust Project Team were aware that the quantitative evaluation of the project was an issue. Evaluation was a challenge due to the length of the project (11 months) and limited funding (the funding pot was very small so that the budget was allocated to the development and production of the intervention tools). The team recognise they would have liked to evaluate further, including involving academic colleagues, however time and funding did not support this. In addition other issues which impacted on the evaluation included prisoners transferred during implementation and evaluation, and reliance on Prison Health Teams to provide data but for whom this was not a priority. Consequently the quantitative results are very limited – including lack of contextual data i.e. there are no records of the numbers of people who took part in the project – but are described in this publication in order to both highlight the possible potential of the intervention but also the issues with the project implementation and evaluation.

### The project evaluation - methodology and findings part 2

This publication initially began as a straightforward description of the development, implementation and evaluation of a project aimed at preventing suicide and self-harm amongst high-risk prisoners. It soon became apparent that, while we could not disguise the issues involved with the roll-out or very limited data available to measure impact, the real results of this project lies in the form of learning for future projects of this nature. Interviews were carried out with three practitioners most closely involved in the project development, delivery and evaluation. Ethics approval for these interviews was granted by the University Ethics Committee. Interviews were semi-structured – interviewees were asked to reflect on the project, its implementation and evaluation. Interviews were tape-recorded and transcribed. Data was analysed using a thematic analysis framework. Six themes were identified including: project focus, communication and professional relationships, the institutional environment, funding and time, roll-out and evaluation, and a ‘champion’ role. These themes are outlined below.

### Project focus

The multi-faceted nature of the project appears to have caused some initial confusion (subsequently resolved) among the project team regarding its focus and coherence, with reflections illustrating that team members had different understandings of the relative importance of each component of the project:

*“There was a (job role) involved in the project at the time who maybe muddied the waters a little bit in terms of what we had to do. There wasn’t clear understanding of what the project actually was. The (job role) was very focused on the trauma-informed stuff…the trauma-informed stuff ended up being quite a small part of the project. I was doing the reading around the trauma informed stuff, but I didn’t see how that linked to the rest of it”. (Interviewee 1).*

Clearly the early stages of the project experienced confusion which is not conducive to effective development and implementation. Reflections also suggest that project team members were given considerable freedom to develop ‘their’ part of the project. The initial project lead decided the content of the in-cell elements of the project based on individual experience, whilst another member of the team focused heavily on the trauma-informed component. Such freedom is positive in that it can promote commitment and engagement. However, reflections suggest it can potentially contribute to a lack of clarity about focus.

### Communication and professional relationships

A key issue emerging from staff reflections is the importance of inter-personal, professional relationships to ensure effective implementation. These relationships are linked to time and institutional issues, discussed in following sections. The project did not enjoy a consistent team of key staff as original team members who developed the project moved on, leading to the aforementioned confusion about the focus of the project. Consequently, as implementation began, a member of the development and delivery team was new into post. This person had no previous experience of working in prisons which, given the timescale for project implementation, made it difficult for them to speedily develop positive relationships, which on reflection, were regarded as crucial to effective implementation:

*“A big part of it is the person working on that project getting to know the prison and some of the staff, so you can go on to a wing and go ‘hi so-and-so do you mind getting a few people around so I can talk about this?’ I was completely new…and I’d never set foot in a prison before…I think having someone with a knowledge of how the prison works and that relationship with the prison teams and the mental health teams makes quite a big difference” (interviewee 1).*

This was problematic as reflections indicate the benefits of having projects staffed by individuals with experience of working in prisons:

*“If you have an idea that’s a little bit different, but they’ve worked with you and you’ve got that relationship where they can ask you frank questions and you can answer them honestly, then people will have some trust in what you’re saying and what you want to do” (interviewee 3).*

Reflections suggest a recognition of the need for explicit promotion activities to engage wider prison staff in projects of this type to support implementation:

*“We should have got more buy in from Officers. We should have sold the product first. You can put the Bob Boxes there, but if they don’t understand what it’s for and how it’ll benefit the prisoners….that was the bit I think we missed” (interviewee 2).*

*“We included the mental health awareness package…the negative is, I’m not sure it has been launched appropriately. I’m not sure how much access there’s been to it….didn’t come in with a big bang. I haven’t had the feeling that bit has happened” (interviewee 3).*

The need to negotiate implementation with the institutions concerned led to its scaling back. For example staff training was initially designed to engage as many staff as possible, but each institution subsequently decided training should be more narrowly focused on specific staff-cohorts:

*“The training, we wanted people of all grades and levels, but the prison decided to have senior officers and maybe some Governors and they’d cascade that information down” (Interviewee 1).*

The benefits of having the project developed by staff with limited experience of working in prisons was recognised, alongside the need to balance this with input from experienced staff to ensure feasibility of ideas. This balance delivered new ideas, whilst maximising the practicality of project content practicality.

*“At the beginning it was good to have someone in the project who didn’t know the prison estate, so they said this might work…they had a blank view. We already have a premeditated view about some things that we think will and won’t work. I would never have thought of Lego but what I said was is to this make this work we need a way to count it in and out. Fresh eyes are important but so is…the experience as well” (interviewee 3).*

However whilst reflections suggest there is a positive contribution to be made by those new to working in prisons, promotion and liaison needs to be undertaken by practitioners with experience of working in prisons:

*“I think it works when you have someone who knows the prison. Now it’s easy for me to go and talk… because I’ve got relationships with the staff on the wings. Having that relationship with the staff is so important so you challenge people…I can do that much better when I’ve got a good relationship with them and do it in a way they don’t think that I’m telling them what they’re thinking is wrong” (interviewee 1).*

### The institutional environment

As discussed, prisons are challenging institutional environments in which to implement new projects. Projects must be implemented within pre-existing policy and practice environments that prioritise security. Staff reflections suggest this environment created difficulties in terms of ensuring optimal project-team access to prisons, speedy roll-out, and agreement about the content of resources developed:

*“I didn’t have security clearance, so was going in ad hoc one or twice a month which was fine for what I was doing, but not enough for what I could have been doing” (interviewee 1).*

*“Security is paramount. So trying to get people cleared, trying to get items cleared, that was a challenge. The equipment we wanted…lego* 2 *is the prime example. Great for distraction…they said no” (interviewee 2).*

Earlier discussion illustrated that prisons have faced considerable challenges in recent years including reduced staffing, alongside a need to address a range of issues. The most recent HMIP inspectorate reports for the institutions concerned contained a total of 105 points to address (HMIP).^3^ Reflections indicate that volume of duties to undertake, alongside the prioritisation of security, created a situation where the project was seen perhaps, by some staff, negatively as another task, rather than as something beneficial for them and prisoners. Indeed, project-staff reflected that perhaps a minority of wider prison staff didn’t see the relevance of the project and so disengaged from it:

*“Most of the staff are great, but it’s just trying to get them on-board and a lot of the time their minds are so fixed on risk and stuff that this kind of stuff falls away….I think the problem is that a new thing comes in and it’s like ‘oh we’ve got another thing to do’” (interviewee 1).*

*“The Mental Health Teams, they weren’t critical, but they weren’t excited. I think they were like ‘this is another thing that we’re going to have to do’, rather than seeing it as actually we’ve got this box of resources” (interviewee 1).*

*“How much they (Safer Custody) truly bought in…there were times when it felt that you were chasing constantly, saying I need you to do this, I need you to do that. I know people are busy. I think we had a few comments by Officers, why are we giving them* Rubik’s *cubes, what’s this about? There was that element of negativity” (interviewee 2).*

The consequence of this partial engagement was that the resources for prisoners do not appear to have been integrated consistently into wider institutional policy and practice to address suicide and self-harm. Some prisoners reported difficulties accessing the boxes, felt that staff were unclear about the purpose of the boxes (although staff feedback suggests otherwise) and that (contrary to the prescribed process) they were not able to choose Bob Box materials themselves.

*“I was in ACCT reviews where they wouldn’t offer resources” (interviewee 1).*

*“(prison staff) were rubbish at it (distributing the Bob Boxes)…I don’t think it was because they thought ‘oh this is stupid’, it’s just because on top of everything else they had to do it’s just extra work” (Interviewee 1).*

It also proved impossible to upload the e-learning packages developed onto the institutional IT systems as the materials and the systems were incompatible. The development of the e-learning resource had not been part of the original project outline, but was developed later as an ‘added bonus’ of the project. The IT challenges had not been foreseen. It was not an issue which arose during meetings between the Trust and the MoJ funders. Initially when problems arose it was assumed that a local solution was possible, however the issue had to be escalated to the national prison IT team.

### Funding and time

Reflections suggest that financial and temporal issues contributed to the difficulties faced by the project. In interviews, project staff discussed their perceptions of a mismatch between project funding, objectives and timescales, which resulted in a scaling-down of the scope of trauma-informed activities.

*“What they* [Ministry of Justice] *wanted us to do actually with the trauma informed service was to develop, implement and evaluate the whole trauma informed service but we went back and said given the timescale and the money you’ve given us it’s not going to happen” (interviewee 1).*

As discussed, a key member of the team was new in post. This situation was exacerbated by a very short hand-over period, resulting in a minimal amount of time for this individual to understand the aims of the project, their roles and responsibilities, and to develop the relationships with other staff that, on reflection, they feel crucial to effective implementation:

*“(name) was leaving a week and half after I came….so a really quick handover. I didn’t know the prisons, didn’t know the service, didn’t know the teams” (interviewee 1).*

Prisons are very hierarchical organisations. As a result it can take time (which the project had a limited amount of) to discuss issues and agree ways forward:

*“To be able to influence you had to go back to the Governors. Lots of layers and structures to go through. We had little mini-project boards. The Governors, this was just part of their role so there were elements of delay in there” (interviewee 2).*

### Roll-out and evaluation

The limitations of delivery methods compromised the roll-out of the project. There was a reliance on engagement from prison staff that reflections suggest was not always forthcoming, whilst the dissemination of information was partially reliant on staff accessing it on the intranet, which was acknowledged as problematic:

*“(information) is now available on the prison intranet. But who’s going to first of all find it in the deep, dark crevices of the intranet?” (interviewee 1).*

Impacts could have been better captured. Although the evaluation methodology included positive elements (e.g. the diagnostic abilities, validity and clinical relevance of the standardised tools have been substantiated) (Donker et al., 2011; Kroenke et al., 2001; Munoz-Navarro et al., 2017; Rossom et al., 2017; Rutter & Brown, 2017; Warwick Medical School, 2013), staff reflections indicate the evaluation was compromised in a number of ways, resulting in it being difficult to determine the impact of the project:

*“In terms of the problem of self-harm and suicide in prisons, this is something that we could have demonstrated does help, but we lost the opportunity to do that…so what impact did it have…hmm…well…?” (interviewee 1).*

Lack of specialist input into evaluation design and focus required staff to develop some tools which, in hindsight, they feel were perhaps inappropriate and failed to capture key information. Mechanisms to capture the longer-term impacts of the project were not integrated into the evaluation methodology. For example, the longer term benefits of staff training were not captured (and a request for follow up funding from the MoJ to enable this was declined). The aforementioned discussion of findings indicates that the evaluation did not systematically collect data from all relevant participants.

*“There wasn’t anyone that had an interest or expertise in research, it just didn’t get considered. We needed input...this is the data you need to be collecting, these are the important things” (interviewee 1).*

*“We should really have planned for the evaluation part of it. What we were going to look at, how we were going to evaluate it and that should have been embedded first” (interviewee 2).*

Staff reflections suggest a possible misallocation of evaluation responsibilities. The reliance on Safer Custody staff to administer and collect prisoner feedback forms was problematic (in that few forms were completed) and time consuming for the project team to oversee:

*“I was emailing safer custody every few weeks to see if they had any (evaluation) forms…they probably hated me by the end of it! It’s just because on top of everything else they had to do it’s just extra work” (interviewee 1).*

*“Ownership seemed to sit within the Safer Custody Teams at both jails. For me it should have probably sat in the Mental Health Team. We (the project team) could’ve owned it more” (interviewee 2).*

### ‘Champion’ role

Interviewees reflected that the crucial relationship between project teams and prison staff could be strengthened by creating a ‘champion’ role for similar, future projects. Their responsibilities would be to explicitly promote and manage wider prison engagement with the project and its evaluation:

*“Maybe having someone from each of the teams (e.g. Safer Custody or Mental Health In-Reach Teams) who is the champion for the project…they could have promoted the resources…they could have gone out and done interviews with those who’ve used Bob Boxes. Go to the ACCT reviews, offer the Bob Box” (Interviewee 1).*

*“The Trauma Informed work….should have been Champions in the workplace who work with offenders face-to-face (interviewee 2)”.*

This role could help to address the issues caused by the delegated responsibility for the administration of in-cell activities. This role could provide the clarity, oversight and key inter-personal relationships that would support prison officer engagement in projects of this kind.

## Discussion

Although there were clearly difficulties which compromised the delivery and evaluation of the project, it is important to acknowledge its positive outcomes. Although limited, initial evaluation findings appear positive. Staff reflections also indicate a positive legacy. This legacy includes continued awareness and use of resources by staff and prisoners, and materials from the project used to further develop practice:

*“Bringing together some tools that don’t need to come directly from mental health is good and to raise understanding of what these tools can do for people” (interviewee 3).*

*“It’s a like a slow-burner…so now in (name of prison) the Mental Health Team uses the packs…the mental health teams give them out all the time which is really positive because it means they’re actually being used” (interviewee 1).*

*“We’ve done a gap analysis looking at trauma informed practice…and we’ve got some training packages now. Something that we’ve done ourselves further down the line” (interviewee 3).*

Perhaps the real legacy of this project is the learning points which emerged from staff reflections of the development, implementation and evaluation and which should be embedded in future similar projects to maximise their chances of success. Not all of these learning points imply radical content or structural change. That said, crucial to a successful delivery model is a budget and timeframe that enable objectives to be achieved. This mismatch undermined any prospect of the project delivering as originally envisaged. It is also disempowering for staff to be given responsibility for the delivery of unrealistic outcomes. Linked to the necessity of appropriate budgets is the need to consider funding sufficient to finance the delivery of a project across multiple sites. This will provide opportunities for more robust evaluation as there will be greater number of participants and contexts, enabling the collection of more useful data. Combined with specialist evaluation input to ensure appropriate focus and use of tools and data collection, this will enable commissioners and practitioners to better understand ‘what works, for whom, in what circumstances, why and how’. Also important is ensuring sufficient time, within project development, to discuss and promote plans to prison staff to maximise their awareness and engagement once implementation begins.

## Conclusions

Interventions to tackle suicide and self-harm in prisons are clearly needed. The reflections by staff involved in the project suggest that staff training and resources for prisoners are potentially helpful. However, reflections also illustrate a number of issues compromised delivery, impacts and evaluation. Recommendations for future projects point to a clear need for: a clear project focus, realistic budgets and timeframes; delivery across multiple sites; the need for specialist evaluation input; awareness and engagement with prison staff; delivery by practitioners who are familiar with prisons; and a ‘champion role’. These issues need to be addressed if projects of this nature, including those focusing on prison suicide and self-harm, are to be effective in the future.

## Abbreviations

ACCT: Assessment, Care in Custody and Teamwork Procedure
GAD-7: Generalised Anxiety Disorder 7
KTP: Knowledge Transfer Partnership
MoJ: Ministry of Justice
PHQ-9: Patient Health Questionnaire 9
PPO: Prison & Probation Ombudsman
WEMWBS: Warwick-Edinburgh Mental Wellbeing Scale
YOI: Young Offenders Institution

## Footnotes

1 The HMIP Inspection Reports are not included in the Reference list to protect the anonymity promised to each institution. Year of publication is also missing to protect anonymity.

2 Lego is now used in some of the region’s prisons as part of follow up activity to the project.

3 See footnote 2.

## References

[CR1] Barker E, Kolves K, De Leo D (2014). Management of Suicidal and Self-Harming Behaviors in prisons: Systematic literature review of evidence-based activities. Archive of Suicide Research.

[CR2] Biggam FH, Power KG (1998). A comparison of problem-solving abilities and psychological distress in suicidal, bullied and protected prisoners. Criminal Justice and Behaviour.

[CR3] Bilderbeck A, Farias M, Brazil I, Jakobowitz S, Wikholm C (2013). Participation in a 10-week course of yoga improves behavioural control and decreases psychological distress in a prison population. Journal of Psychiatric Research.

[CR4] Brooker C, Flynn J, Fox C (2010). Trends in self-inflicted deaths and self-harm in prisons in England and Wales (2001–2008): In search of a new research paradigm.

[CR5] Crighton DA, Towl GJ (1998). Suicides in prison in England and Wales. Prison Service Journal.

[CR6] Daigle M, Daniel A, Dear G, Frottier P, Hayes L, Kerkhof A, Konrad N, Liebling A, Sarchiapone M (2007). Preventing suicide in prisons, part II. Crisis.

[CR7] Daniel A (2006). Preventing suicide in prison: A collaborative responsibility of administrative, custodial and clinical staff. The Journal of the American Academy of Psychiatry and the Law.

[CR8] Dear, G. (1999) ‘Preventing Self-harm in Prison: The Need to Address Situational Factors that Give Rise to Distress’, paper presented at the Australian and new Zealand Society of Criminology 14th annual National Conference, Perth.

[CR9] Donker, T et al (2011) ‘Quick and easy self-rating of generalized anxiety disorder: Validity of the Dutch web-based GAD-7, GAD-2 and GAD-SI’, Psychiatry Research, 30 June 2011, Vol.188 (1), pp.58–64. 10.1016/j.psychres.2011.01.016 21339006

[CR10] Eccleston L, Sorbello L (2002). The RUSH program – Real understandings of self-help. A suicide and self-harm prevention project within a prison setting. Australian Psychologist.

[CR11] Foster, J (2011) ‘Peer support in prison healthcare, an investigation into the listening scheme in one adult male Prison’. London: University of Greenwich.

[CR12] Havenger, S (2010) Trauma-Informed Care and Practice at http://www.mhcc.org.au/project/trauma-informed-care-and-practice-ticp/x. Accessed 23 Nov 2018.

[CR13] Hawton K, et al (2014) ‘Self-harm in prisons in England and Wales: an epidemiological study of prevalence, risk factor, clustering and subsequent suicide’. *The Lancet Online Publication*, *383*(*9923*),1147–1154. 10.1016/S0140-6736(13)62118-2 PMC3978651 24351319

[CR14] Hayes AJ, Shaw J, Lever-Green G, Parker D, Gask L (2008). Improvements to suicide prevention training for prison staff in England and Wales. Suicide and Life Threatening Behaviour.

[CR15] Humber N, Hayes A, Senior J, Fahy T, Shaw J (2011). Identifying, monitoring and managing prisoners at risk of self-harm/suicide in England and Wales. The Journal of Forensic Psychiatry and Psychology.

[CR16] Kaster T, Martin M, Simpson A (2017). Preventing Prison Suicide with Life Trajectory-Based Screening. The Journal of the American Academy of Psychiatry and the Law.

[CR17] Konrad N, Daigle M, Daniel, Dear G, Frottier P, Hayes L, Kerkhof A, Liebling A, Sarchiaphone M (2007). Preventing suicide in prisons, part 1. Crisis.

[CR18] Kroenke, K., Spitzer, R. and Williams, J., (2001) ‘The PHQ-9: Validity of a brief depression severity measure’, Journal of General Internal Medicine 2001 Sept 16 (9):606–613. 10.1046/j.1525-1497.2001.016009606.x PMC1495268 11556941

[CR19] Leese M, Thomas S, Snow L (2006). An ecological study of factors associated with rates of self-inflicted death in prisons in England and Wales. International Journal of Law and Psychiatry.

[CR20] Lutz SJ (1990). The effect of relaxation training on sleep, state anxiety, and sick call in a jail population. Journal of Prison & Jail Health.

[CR21] Maranzo L, Hawton K, Rivlin A, Smith EN, Piper M, Fazel S (2016). Prevention of Suicide Behavior in Prisons. Crisis.

[CR22] Marzano L, Ciclitira K, Adler J (2012). The impact of prison staff responses on self-harming behaviours: Prisoners’ perspectives. The British Journal of Clinical Psychology / the British Psychological Society.

[CR23] Miller NA, Najavits LM (2012). Creating trauma informed correctional care: A balance of goals and environment. European Journal of Psychotraumatology.

[CR24] Ministry of Justice (2012). Management of prisoners at risk of harm to self, to others and from others. Prison Service Order 2700. Prison Service Instruction 64/2011.

[CR25] Ministry of Justice (2013). Management of prisoners at risk of harm to self, to others and from others (Safer Custody).

[CR26] Ministry of Justice (2016). Management of prisoners at risk of harm to self, to others and from others (Safer Custody) PSI 64/2011.

[CR27] Ministry of Justice (2017). Safety in custody statistics, England and Wales: Deaths in Prison Custody to September 2017 Assaults and Self-Harm to June 2017.

[CR28] Ministry of Justice (2018). Safety in custody statistics, England and Wales: Deaths in Prison Custody to December 2017 Assaults and Self-harm to September 2017.

[CR29] Munoz-Navarro R (2017). Screening for generalized anxiety disorder in Spanish primary care centers with the GAD-7. Psychiatry Research.

[CR30] National Audit Office (2017). Mental Health in Prisons.

[CR31] National Resource Centre for Justice Involved Women (2013) Innovators: Lynn Bissonnette, Massachusetts Correctional Institution – Framingham. Retrieved from https://cjinvolvedwomen.org/massachusetts-correctional-institution-at-framingham/. Accessed 23 Nov 2018.

[CR32] Naud H, Daigle M (2013). How to improve testing when trying to predict inmate suicidal behaviour. International Journal of Law and Psychiatry.

[CR33] Patry MW, Magaletta PR (2014). Measuring suicidality using the personality assessment inventory: A convergent validity study with Federal Inmates. Assessment.

[CR34] PPO (Prison & Probation Ombudsman) (2017) Annual Report, 2016–17.Crown Copyright.

[CR35] PPO (Prisons & Probation Ombudsman) (2014). Annual Report 2013–2014.

[CR36] PPO (Prisons & Probation Ombudsman) (2015). Annual Report 2014–2015.

[CR37] Prison Service Instruction (PSI) 64 (2011). Management of prisoners at risk of harm to self, to others and from others (safer custody), Ministry of Justice.

[CR38] Prisons & Probation Ombudsman (2016) Learning from PPO investigations: Self-inflicted deaths of prisoners on ACCT. Crown copyright.

[CR39] Ramluggun P (2011). Beyond observation: Self-harm in prisons. Mental Health Practice.

[CR40] Rethink Mental Illness (2014). Factsheet, Prisoners – Suicidal Thoughts.

[CR41] Rossom R (2017). Suicidal ideation reported on the PHQ9 and risk of suicidal behavior across age groups. Journal of Affective Disorders.

[CR42] Rutter, L & Brown, T (2017) ‘Psychometric properties of the generalized anxiety disorder Scale-7 (GAD-7) in outpatients with anxiety and mood disorders’, Journal of Psychopathology and Behavioral Assessment, 2017, Vol.39(1), pp.140–146. 10.1007/s10862-016-9571-9 PMC5333929 28260835

[CR43] Sakinofsky I (2014). Preventing suicide among inpatients. Canadian Journal of Psychiatry.

[CR44] Shapiro D, Cline K (2008). Mood changes associated with iyengar yoga practices: A pilot study. International Journal of Yoga Therapy.

[CR45] Shaw J, Baker D, Hunt IM, Moloney A, Appleby L (2004). Suicide by prisoners: A national clinical survey. British Journal of Psychiatry.

[CR46] Steel J, Thornicroft G, Birmingham L (2007). Prison mental health in reach services. British Journal of Psychiatry.

[CR47] Timmerman IGH, Emmelkamp PMG (2001). The relationship between traumatic experiences, dissociation, and borderline personality pathology among male forensic patients and prisoners. Journal of Personality Disorders.

[CR48] Warwick Medical School (2013) ‘Strengths of WEMWBS’, at : https://warwick.ac.uk/fac/sci/med/research/platform/wemwbs/development/strengths/. Accessed 23 Nov 2018.

[CR49] Wolff N, Huening J, Shi J, Frueh BC (2014). Trauma Exposure and Posttraumatic Stress Disorder among Incarcerated Men. Journal of Urban Health: Bulletin of the New York Academy of Medicine.

[CR50] World Health Organisation (2007). Preventing Suicide in Jails and Prisons.

[CR51] Yoshihara K, Hiramoto K, Sudo N, Kubo C (2011). Profile of mood states and stress-related biochemical indices in long-term yoga practitioners. Biopsychosocial Medicine.

